# Reward Responsiveness, Optimism, and Social and Mental Functioning in Children Aged 6-7: Protocol of a Cross-Sectional Pilot Study

**DOI:** 10.2196/18902

**Published:** 2020-09-24

**Authors:** Charlotte Vrijen, Mégane Alice Ackermans, Anna Bosma, Tina Kretschmer

**Affiliations:** 1 Faculty of Behavioural and Social Sciences University of Groningen Groningen Netherlands

**Keywords:** optimism, reward responsiveness, risk-taking, children, mental health, social relations

## Abstract

**Background:**

There is evidence that reward responsiveness and optimism are associated with mental and social functioning in adolescence and adulthood, but it is unknown if this is also the case for young children. Part of the reason for this gap in the literature is that the instruments that are used to assess reward responsiveness and optimism in adolescents and adults are usually not suitable for young children.

**Objective:**

Two behavioral tasks to assess reward learning, a questionnaire on reward responsiveness, and a questionnaire on optimism/pessimism will be tested on their feasibility and reliability in children aged 6-7. Depending on their feasibility and reliability, these instruments will also be used to investigate if reward responsiveness and optimism are associated with mental and social functioning in young children.

**Methods:**

For this cross-sectional pilot study, we adapted a number of tasks and questionnaires to the needs of 6-7-year-old children, by simplification of items, oral rather than written assessment, and reducing the number of conditions and items. We will approach teachers and, with their help, aim to include 70 children aged 6-7 to assess the feasibility and reliability of the tasks and questionnaires. Feasibility measures that will be reported are the proportion of children completing the task/questionnaire, the proportion of children that were able to explain the instructions in their own words to the researcher, and the proportion of children that correctly answered the control questions. The reliability of the scales will be assessed by computing Cronbach α and item-total score correlations and the reliability of the tasks by correlations between different consecutive blocks of trials. Ethics approval was obtained from the Ethics Committee of the Department of Pedagogy and Educational Sciences.

**Results:**

Data collection was originally planned in March and April 2020, but has been postponed due to Corona virus regulations. We expect to collect the data in the first half of 2021. The findings will be disseminated in preprints and peer-reviewed publications.

**Conclusions:**

The development of feasible and reliable instruments for assessing reward responsiveness and optimism in young children is expected to benefit future research on underlying mechanisms of mental and social functioning in young children. If the instruments assessed in this study are usable with young children, it would be particularly interesting to include them in cohort studies because this would enable investigating not only concurrent associations, but also prospective associations between reward responsiveness and optimism early in life and mental and social functioning later in life. If, as we hypothesize, reward responsiveness and optimism are not only associated with (prospective) mental and social functioning in adults and adolescents but also in young children, this could provide a way of identifying vulnerable children already at an early stage.

**International Registered Report Identifier (IRRID):**

PRR1-10.2196/18902

## Introduction

### Background

Research suggests that reward responsiveness and optimism are important for mental and social functioning in adults and adolescents [1-8]. Rewards play an important role in shaping our behavior in everyday life [9,10]. Rewards can be very diverse, for instance, monetary (eg, winning the lottery), winning a game, eating one’s favorite meal, and social rewards (eg, being liked or receiving a compliment from a friend). People in general adapt their behavior in the presence or prospect of a reward, with the aim of maximizing the reward or increasing the chance of future rewards. That is, individuals learn from the conditions in which they receive rewards and adapt their behavior accordingly [11,12]. For example, if a certain strategy results in gaining points in a game, being liked by others, or receiving social praise, individuals will start using this strategy more often. This is a specific type of reward responsiveness, commonly referred to as *reward learning*. Although people generally respond strongly to rewards, individual differences can be observed with respect to the strength of the response. These differences can be important as there is ample evidence that low reward responsiveness is associated with current depression [1,2,13] and prospectively predicts depression [14-17]. Very high reward responsiveness is associated with other types of mental health problems, for example, addiction and criminal behavior [3,18]. There is also some evidence suggesting that reward responsiveness is associated with social functioning [17,19], that is, individuals with higher reward responsiveness show higher levels of sociability and emotional intelligence [19], and report higher friendship quality [17].

Not only reward responsiveness, but also optimism is important for mental and social functioning in adults and adolescents [4,20,21]. Optimism refers to the general belief that things will go well and that the future will turn out good rather than bad [5]. Optimists, when confronted with a setback, believe that this is not indicative of a personal weakness and are motivated to overcome the problem. Pessimists, by contrast, are more likely to have negative expectations about themselves and the people around them and are more likely to give up when faced with challenges. Compared to pessimists, optimists report higher levels of subjective well-being, better mental health, and are more liked by other people [6,22]. A more optimistic attitude toward life is, for example, associated with lower levels of depressive symptoms [6,21] and optimists are more socially accepted than those who show a less optimistic view on life [7,23]. More optimistic college freshmen experience more social support and report larger friendship networks [8]. However, unrealistic optimism has been found to be related to harmful risk-taking behavior [24].

There is evidence that reward responsiveness and optimism are associated with mental and social functioning in adolescence and adulthood, but it is unknown if this is also the case for young children. Part of the reason for this gap in the literature is that the assessment methods that are used for adolescents and adults are usually not suitable for young children. Another gap in the literature is the lack of knowledge about the relation between reward responsiveness and optimism. It seems plausible that high reward responsiveness extends to expectations about the future in that positive rather than negative information may primarily be used to form future expectations, which is characteristic of an optimistic view on life. Optimists tend to update their beliefs less based on negative experiences or information that is more negative than expected and update their beliefs more based on rewarding experiences or positive information [24,25]. Optimism may also lead to higher attention to rewards and lower attention to the negative aspects of life, even in stressful or difficult situations. However, to our best knowledge, associations between reward responsiveness and optimism have not been investigated directly, let alone in young children.

### This Study

For this pilot study, we adapted a number of tasks and questionnaires to the needs of 6-7-year-old children, by reformulating questionnaire items, using oral rather than written assessment, and reducing the number of experimental conditions and questionnaire items. The adapted instruments will be piloted for feasibility, and will be used to investigate if reward responsiveness and optimism are associated with mental and social functioning already in young children. Associations between reward responsiveness and optimism will also be investigated. Importantly, if the new instruments work for young children, this could benefit many different types of future studies. It would be particularly interesting to include the instruments in cohort studies to facilitate investigating prospective associations. This would mean that, in time, we would be able to investigate whether reward responsiveness and optimism at age 6-7 prospectively predict mental and social functioning later in life. This information may ultimately inform preventive programs aimed at modifying reward responsiveness and optimism in early to middle childhood.

### Research Questions

1. Do 6-7-year-old children understand the tasks and questionnaires and is the length of the tasks and questionnaires feasible for this young age group?

2. What is the reliability (ie, internal consistency) of the instruments?

3. Are individual differences in aspects of reward learning during the reward tasks associated with each other and with self-report measures of reward responsiveness and optimism in 6-7-year-old children? Reward learning outcomes are, for example, how fast children learn from the conditions in which they receive a reward and the extent to which they are willing to take risks to earn a reward.

4. Are individual differences in reward learning, reward responsiveness, and optimism associated with mental functioning (teacher report) and social functioning (teacher and classmate reports) in 6-7-year-old children?

## Methods

### Sample and Procedure

We aim to include 70 children aged 6-7. A linear regression power analysis in G*Power 3.1 [26] showed that, with power set to 0.80 and α to .05, a minimum of 55 participants is required to find a moderate effect. A similar number of participants is required for a moderate correlation. An additional 15 participants will be recruited to compensate for possible drop out. Because of the explorative nature of this pilot study, we will not correct for multiple testing.

Complete school classes will be recruited (group 3 in the Dutch school system) via the teachers. Depending on the size of the classes and the number of parents willing to give consent for their children to participate in the study, we expect to include 4-7 school classes. Teachers will be approached by telephone, email, and social media. They will receive an information letter and a consent form. After a teacher has signed the consent form, the parents of the children in the class will be contacted. They will also receive an information letter and consent form. Only when the teacher and parents of a child give consent, the child will be included in the study. The child will be asked to give informed consent orally on the day of the assessment and will be assured that he/she can stop at any time during the assessment. Ethics approval was obtained from the Ethics Committee of the Department of Pedagogy and Educational Sciences of the University of Groningen (ref. 04032020).

Child assessment consists of 2 computer tasks and 3 short questionnaires (18 items in total). Children will be assessed individually in a separate room by a researcher who reads all instructions and items to the child, checks if the child understands them, and remains present during the entire assessment. After the final task, the researcher will ask the child what he or she liked and disliked about the tasks and if everything was clear. Additionally, for children who disclosed bullying, the researcher will ask if the child already talked about this with a parent, teacher, or other adult. If not, the child will be encouraged to do so and the researcher will offer to help with this. The estimated total duration of the child assessment is 30-40 minutes. Children will receive 3 stickers and a small present.

Teachers will receive a weblink to answer questions about the mental (8 items) and social functioning (9 items) of the participating children in their classroom, with an expected duration of 5-10 minutes per child. Teachers will receive a gift card of €40 (US $47) for participating in the study to purchase something for the class. Teachers will be debriefed individually by a researcher after all child assessments and online questionnaires are completed. Teachers of classes in which bullying was disclosed will be encouraged to discuss bullying in the classroom or with children individually, if they have not done so already. For privacy reasons, no information about experiences of specific children will be shared with teachers. The tasks, questionnaires, and feasibility assessments will be described in detail per task in the “Measures” section.

Data will be stored on institutional network drives with security firewalls and access to the data will be limited to the study team. Data will be kept deidentified and a password-protected file with identifiers will be stored separately from the data. Raw data will not be shared publicly for reasons of privacy (ie, this would enable coupling children with certain unique task scores to teacher reports), but researchers interested in the data can submit a research proposal to the first author (CV) of this study protocol.

### Measures

#### Reward Learning Tasks

Two tasks will be used to assess reward learning. Because it is possible that experience with one of the tasks can influence performance on the next task, half of the children in each class will start with one task and the other half with the other task. The second task will not be assessed directly after the first one, but only after a block of questions. Each child will be assessed individually by a researcher, who will score whether the child is able to explain the instructions in his/her own words to the researcher, and whether the child correctly answers the control questions asked by the researcher.

##### Task 1: Probability Learning Task: Finding Gold Coins (PL Gold Coin Task)

To assess reward learning, a computer task that has the appearance of a computer game will be used. The child is instructed to search for gold coins that have been hidden by an elf under 1 out of 6 rocks. Children are instructed to click with the mouse on the rock under which they think the gold coin is hidden. After choosing a rock, the child is shown if the coin is hidden there, and, if not, is shown the correct location of the coin. Every time the elf appears the child can search for a new gold coin, 120 times in total. Unknown to the child, the gold coins will be hidden under the same rock in 75% (90/120) of the cases, there are 2 rocks under which the coin is hidden in 10% (12/120) of the case, 1 rock under which the coin is hidden in 5% (6/120) of the cases, and 1 rock under which the coin is never hidden. The task assesses to what extent children adapt their search strategy to the most frequent location of the coin.

The task was originally developed by Plate and colleagues [27], who gave permission for its use and adaptation. The original task consisted of 8 rocks, 200 trials, and the largest proportion of gold coins under the same rock was 70%. The number of trials was too high for 25% of the children between ages 4 and 11 and for these children the data could not be used [27]. Therefore, we shortened the task to 120 trials, decreased the number of rocks from 8 to 6, and increased the largest proportion of gold coins under the same rock from 70% to 75%. We expect that these changes will ensure that children learn faster from the rewards than in the original task and that our shorter version of the task is still sufficient to assess individual differences in reward learning. A final adaptation to the original task is that the location of the rock with the largest proportion of gold coins will be varied among children. In the original task, this rock was always in the same location, somewhere in the middle, and we want to prevent that choosing the middle rock more often than the more peripheral rocks is unjustly interpreted as a learning effect when it is actually a preference for central locations that is unrelated to reward learning. All of the changes we made to the original task were discussed with the researcher who developed the task.

The task is programmed in E-Prime 2 and will be assessed on a laptop. After 60 trials, the child is given a short break. The estimated total duration of the task, including instruction and break, is 12 minutes. After the instruction and before the actual task starts, the researcher checks whether the child understands the task by asking the child to explain the task to the researcher. If this is too difficult, the researcher asks the following more specific questions: *How do you search for gold coins?,* *What do you see on the screen if you have found a gold coin under a rock?,* and *What do you see on the screen if you picked the wrong rock?*

At the start of the task, the children are told that they get to choose a present from 1 of 3 boxes after completing the task. They are told they can choose a present from box 1 if they find a small number of coins, a somewhat larger present from box 2 if they find more coins, and a large present from box 3 if they find a large number of coins. It is part of the task that the children believe that the amount of gold coins they find determines the size of the present they receive. Unknown to the children, at the end of the task all of them will be told that they did a good job and can choose a present from box 3.

##### Task 2: The Balloon Emotional Learning Task (BELT)

In this task, a small colored balloon appears on the computer screen. The children are instructed that they can pump up the balloon by pressing the space bar. The more often they press the space bar, the more the balloon inflates. The larger they pump up the balloon, the more points the child earns, but no points are earned if the balloon pops. The child is told that some balloons are stronger than others and can be pumped up further before they pop. After each successful pump, the child needs to choose between pumping up the balloon further and pressing enter to cash the points earned for that specific balloon. If the balloon pops before the points are cashed, the child loses the points for that specific balloon. The tests consist of 45 balloons in total. Unknown to the children, the 3 different colors of the balloons represent their strength: the orange balloon always pops after 7 pumps, the pink balloon always pops after 19 pumps, and the blue balloon has a variable strength, popping after 7, 13, or 19 pumps. The task assesses how much risk the child is willing to take to obtain a larger reward and the extent to which the child learns from feedback about the circumstances (here, color of the balloon) under which risk taking results in reward and the circumstances under which it does not [28].

This task was developed by Humphreys and colleagues [28] and was used in children as young as 3 years of age. The original task consists of 29 balloons (trials), but the researchers themselves suggested that it would be better to use a longer task in future research [28]. We received permission to use the task and increased the number of trials from 29 to 45.

The task is programmed in E-Prime 2 and will be assessed on a laptop. Stickers will be used to make it easier for young children to remember the keys they need to use. That is, on the space bar, they will see a sticker of a balloon and on the enter key a picture of a prize meter, similar to the one they see on the screen. The estimated total duration of the task, including instruction, is 5 minutes. After the instruction and before the actual task starts, the researcher checks whether the child understands the task by asking the child to explain the task to the researcher. If this is too difficult, the researcher asks the following more specific questions: *How do you pump up a balloon?* or *What do you do if you want to stop pumping and save the points you’ve earned?*

To motivate the children to do their best, they are told that if they gain enough points they can choose 2 stickers at the end of the task. Unknown to the children, at the end of the task all of them will be told that they did a good job and can choose 2 stickers.

#### Child Questionnaire Measures

Similar to the tasks, each child will be assessed individually. A researcher will ask questions about the child’s emotional responses to rewards, optimism/pessimism, and social experiences in the classroom. All instructions and questions will be read to the child and the researcher will enter the answers in Qualtrics (Qualtrics Inc.).

##### Pleasure Scale to Assess Emotional Responses to Rewards

To assess children’s emotional responses to rewards, an adapted and shortened version of the Children’s Pleasure Scale by Kazdin [29] will be used. We assess how happy a child feels if situations occur that are commonly perceived as pleasurable (eg, receiving a present, or a compliment). Children will be asked to rate each situation on a 3-point Likert scale and indicate if the situation would make them (1) very happy, (2) happy, or (3) it would not matter. A large card depicting 3 smileys (from very happy to neutral) paired with the 3 response options will be placed in front of the child and the child can either answer by talking or by pointing to one of the smileys. Before beginning the actual interview, the researcher checks if the child understands the response options by presenting the 3 response options to the child orally, in a different order than in the original explanation, and asking the child to point at the picture belonging to the specific response. If the child makes a mistake for one of the response options, all 3 are repeated until the child shows the correct response for all options. The child is also asked to respond to an example item (ie, *You get to eat a piece of candy you like*).

The original questionnaire has been used with 6-year-old children [29], but was not validated specifically for a young age group. Some questions seem rather complicated or not applicable to our young age group (ie, *You accidentally overhear your teacher bragging to the principal about what a terrific student you are*). With 39 items, the original questionnaire is also very long. Therefore, we simplified and shortened the original questionnaire and translated it into Dutch. In detail, our research team, including a former primary school teacher (AB) who has experience with our specific age group, selected 10 items based on high item-total score correlations [29], relevance for our age group, and low level of difficulty. These items were translated, simplified (if necessary), and tested in a pilot. Five parents trialed the revised items with their 6-7-year-old children and reported that all of the children were able to answer the questions and indicated that the answers matched with what they would have expected their child to answer. Finally, we selected the 8 most relevant items with the least overlap, while retaining all 3 pleasure domains from the original questionnaire, that is, the domains of physical pleasure (ie, eating a favorite meal), social pleasure (ie, being told what a good friend you are), and other types of pleasure (ie, winning a game). See Multimedia Appendix 1 for the original items and the (translated) reformulated items.

##### Optimism/Pessimism

Optimism/pessimism will be assessed with an adapted and shortened version of the Youth Life Orientation Test (YLOT) [30,31]. Children are presented with different statements and are asked how often they think this: never, sometimes, often, or all the time. Following the procedure of Bamford and Lagattuta [31], a large card depicting 4 boxes ranging from empty to full, paired with the 4 response options, will be placed in front of the child. The child can answer by either talking or pointing to one of the boxes. Before beginning the actual interview, the researcher checks if the child understands all response options. The child is also asked to respond to 2 example items (ie, *I think fries are tasty* and *I like eating worms*).

The original questionnaire has been validated only among children aged 8 and older [30], consists of several questions about the future that are likely too difficult for 6-7-year olds, and was long (19 items). Based on a version adapted for and used with 6-year olds [31], but which still contained several difficult items (ie, *I’m always hopeful about my future*), we created a further simplified and shortened Dutch version. We followed a similar procedure as for the Children’s Pleasure Scale. We first selected 8 items based on high item-total score correlations [30], relevance for the targeted age group and low level of difficulty. These items were translated and further simplified if necessary. Five parents trialed these questions with their 6-7-year-old children. Four of them reported back to us that their child had difficulties understanding the item *Usually, I don’t think good things will happen to me,* therefore, this item was excluded. Parents reported that their children were able to answer the other questions and that the answers matched with what they would expect their child to answer. Finally, we selected the 6 most relevant items by dropping one more item because of overlap with another item. See Multimedia Appendix 1 for the original items and the (translated) reformulated items.

##### Social Experiences

To assess peer acceptance and rejection, children will be asked to nominate the children in their classroom they like to play with and the ones they do not like to play with. Peer aggression and peer victimization will also be assessed using peer nominations. Because of the wide range of reading abilities at ages 6 to 7, photographs are used to facilitate peer nomination in this young age group [32-34]. Children will be presented with a list including the names and photographs of the children in their classroom whose parents have consented, and are asked to either state the names or point to the pictures. The photographs will only be used during the assessment and will be destroyed directly afterward. For all peer nomination measures, proportion scores will be calculated (number of nominations/[number of participating children in the classroom – 1]); this is the gold standard for assessing acceptance and rejection [32].

The use of peer nominations with children sometimes raises the question of whether being assessed with these instruments may have negative consequences for the children. However, studies among children and their teachers suggest that children hardly show negative emotional responses after the use of peer nominations similar to the ones we will use, and also that children are not treated differently in the classroom afterward [35]. The youngest children in these studies were 8 years old, only slightly older than our sample, and we have no reasons to assume that results are different for our age group.

#### Teacher Questionnaires on Children’s Mental and Social Functioning

##### Mental Functioning

Mental functioning of the children will be assessed with the Teacher’s Checklist of Psychopathology (TCP). The TCP is a shortened version of the Teacher’s Report Form [36] and was developed for the TRacking Adolescents’ Individual Lives Survey (TRAILS) [37]. The TCP assesses 9 problem domains: Withdrawal, Somatic complaints, Anxious/depressed symptoms, Social problems, Thought problems, Attention problems, Activity/impulsivity, Aggressive behavior, and Delinquent behavior. The checklist includes descriptions of the corresponding problem behavior for each of these domains. Because we did not have a specific research interest in associations between reward responsiveness/optimism and somatic complaints, this domain (Somatic complaints) was excluded. For the domain Delinquent behavior, we removed parts of the description that were not age appropriate, for example, pertaining to skipping classes and using alcohol or drugs. (For the specific items, see Multimedia Appendix 2.)

##### Social Functioning

In addition to the classmates, teachers will also be asked about the children’s social functioning. Whereas classmates can observe their peers’ more subtle behaviors that may remain hidden for parents and teachers, teachers can provide an accurate general perspective on children’s social functioning that is complementary to the perspective of classmates [35], and it has been advised to combine the 2 complementary perspectives [32]. Teachers will complete a questionnaire on peer aggression, peer victimization, and acceptance (9 items), as used in the Quebec Newborn Twin Study [38], for each of their students participating in the study (see Multimedia Appendix 2).

### Statistical Analysis Plan

The feasibility of the tasks and questionnaires for 6-7-year-old children will be determined by calculating for each task and questionnaire the number of children that completed it, the number of children that were able to explain the instructions in their own words to the researcher, and the number of children that correctly answered the control questions of the researcher prior to the assessment. The reliability of the scales assessing emotional responses to reward and optimism/pessimism will be determined by calculating Cronbach α. Item-total score correlations will also be reported. The reliability of the tasks will be assessed by calculating correlations between different consecutive blocks. Split-half reliabilities will not be computed, because we expect children to learn during the tasks and, therefore, would not expect the first and second half of a task to be highly correlated.

Although we aim to investigate relations between reward responsiveness and optimism and mental and social functioning in 6-7-year-old children, it depends on the feasibility and reliability of the child questionnaires and tasks if statistical tests of these associations are meaningful. In any case, we will report descriptive statistics for all measures. For all scales, sum scores will be reported. The following task outcomes will be reported: the number of points earned during the Probability Learning (PL) Gold Coin Task and the Balloon Emotional Learning Task (BELT), and the number of pumps (general risk taking) [39] and explosions (uncontrolled risk taking) during the BELT [39]. All BELT outcomes will be reported separately for the 3 different conditions (ie, colors). For both tasks, these outcomes will be reported for the task in total, as well as separately for the different blocks.

## Results

Data collection was originally planned in March and April 2020, but has been postponed due to Corona virus regulations. We expect to collect the data in the first half of 2021. Results will be disseminated in deidentified and aggregated form in one or more preprints and peer-reviewed publications. Main findings of this study will be shared on social media and teachers who participate in the study will receive a report with the main findings. Additionally, if the tasks and questionnaires are usable for young children, they will be considered for inclusion in the next wave of the large intergenerational cohort study TRAILS Next [40].

## Discussion

We will investigate the feasibility and reliability of tasks and questionnaires assessing reward learning, reward responsiveness, and optimism specifically for 6-7-year-old children, an age group often assessed in longitudinal birth-cohort studies. Our findings could benefit many researchers interested in studying reward responsiveness and optimism in young children. If the instruments assessed in this study are usable with young children, it would be particularly interesting to include them in cohort studies because this would enable investigating prospective associations between reward responsiveness and optimism early in life and mental and social functioning later in life, and may, ultimately, provide a way of identifying vulnerable children already at an early stage. Because of the exploratory nature of this pilot study, we do not correct for multiple testing, thus any results we find about associations between the different measures are tentative, awaiting replication in a different sample.

## Appendix

Multimedia Appendix 1 All child questionnaire items used in the present study, including the Dutch translations and original formulations.

Multimedia Appendix 2 All teacher questionnaire items used in the present study, including the Dutch translations and original formulations.

## Abbreviations

BELT: Balloon Emotional Learning Task
PL: probability learning
TCP: Teacher’s Checklist of Psychopathology
TRAILS: TRacking Adolescents’ Individual Lives Survey
YLOT: Youth Life Orientation Test

## References

[1] Forbes EE, Dahl RE (2012). Research Review: altered reward function in adolescent depression: what, when and how?. J Child Psychol Psychiatry.

[2] Luking KR, Pagliaccio D, Luby JL, Barch DM (2016). Reward Processing and Risk for Depression Across Development. Trends Cogn Sci.

[3] Hasking PA (2007). Reinforcement sensitivity, coping, and delinquent behaviour in adolescents. J Adolesc.

[4] Ji JL, Holmes EA, Blackwell SE (2017). Seeing light at the end of the tunnel: Positive prospective mental imagery and optimism in depression. Psychiatry Res.

[5] Carver CS, Scheier MF, Segerstrom SC (2010). Optimism. Clin Psychol Rev.

[6] Patton GC, Tollit MM, Romaniuk H, Spence SH, Sheffield J, Sawyer MG (2011). A prospective study of the effects of optimism on adolescent health risks. Pediatrics.

[7] Carver CS, Kus LA, Scheier MF (1994). Effects of Good Versus Bad Mood and Optimistic Versus Pessimistic Outlook on Social Acceptance Versus Rejection. Journal of Social and Clinical Psychology.

[8] Brissette I, Scheier MF, Carver CS (2002). The role of optimism in social network development, coping, and psychological adjustment during a life transition. J Pers Soc Psychol.

[9] Clark A (2013). Reward processing: a global brain phenomenon?. J Neurophysiol.

[10] Mazur JE (2016). Learning & Behavior (Eighth Edition).

[11] Schultz W (2015). Neuronal Reward and Decision Signals: From Theories to Data. Physiol Rev.

[12] de Wit S, Dickinson A (2009). Associative theories of goal-directed behaviour: a case for animal-human translational models. Psychol Res.

[13] Pizzagalli DA, Iosifescu D, Hallett LA, Ratner KG, Fava M (2008). Reduced hedonic capacity in major depressive disorder: evidence from a probabilistic reward task. J Psychiatr Res.

[14] Vrijen C, Hartman CA, Oldehinkel AJ (2016). Slow identification of facial happiness in early adolescence predicts onset of depression during 8 years of follow-up. Eur Child Adolesc Psychiatry.

[15] Vrijen C, Hartman CA, Oldehinkel AJ (2019). Reward-Related Attentional Bias at Age 16 Predicts Onset of Depression During 9 Years of Follow-up. J Am Acad Child Adolesc Psychiatry.

[16] Bress JN, Foti D, Kotov R, Klein DN, Hajcak G (2013). Blunted neural response to rewards prospectively predicts depression in adolescent girls. Psychophysiology.

[17] Rawal A, Collishaw S, Thapar A, Rice F (2013). 'The risks of playing it safe': a prospective longitudinal study of response to reward in the adolescent offspring of depressed parents. Psychol Med.

[18] van Hemel-Ruiter ME, de Jong PJ, Oldehinkel AJ, Ostafin BD (2013). Reward-related attentional biases and adolescent substance use: the TRAILS study. Psychol Addict Behav.

[19] Bacon AM, Corr PJ (2017). Motivating emotional intelligence: A reinforcement sensitivity theory (RST) perspective. Motiv Emot.

[20] Holmes EA, Blackwell SE, Burnett Heyes S, Renner F, Raes F (2016). Mental Imagery in Depression: Phenomenology, Potential Mechanisms, and Treatment Implications. Annu Rev Clin Psychol.

[21] Puskar KR, Sereika SM, Lamb J, Tusaie-Mumford K, McGuinness T (1999). Optimism and its relationship to depression, coping, anger, and life events in rural adolescents. Issues Ment Health Nurs.

[22] Forgeard M, Seligman M (2012). Seeing the glass half full: A review of the causes and consequences of optimism. Pratiques Psychologiques.

[23] Helweg-Larsen M, Sadeghian P, Webb MS (2002). The Stigma of Being Pessimistically Biased. Journal of Social and Clinical Psychology.

[24] Sharot T (2011). The optimism bias. Curr Biol.

[25] Sharot T, Korn CW, Dolan RJ (2011). How unrealistic optimism is maintained in the face of reality. Nat Neurosci.

[26] Faul F, Erdfelder E, Buchner A, Lang A (2009). Statistical power analyses using G*Power 3.1: tests for correlation and regression analyses. Behav Res Methods.

[27] Plate RC, Fulvio JM, Shutts K, Green CS, Pollak SD (2018). Probability Learning: Changes in Behavior Across Time and Development. Child Dev.

[28] Humphreys KL, Telzer EH, Flannery J, Goff B, Gabard-Durnam L, Gee DG, Lee SS, Tottenham N (2016). Risky decision making from childhood through adulthood: Contributions of learning and sensitivity to negative feedback. Emotion.

[29] Kazdin AE (1989). Evaluation of the Pleasure Scale in the assessment of anhedonia in children. J Am Acad Child Adolesc Psychiatry.

[30] Ey S, Hadley W, Allen DN, Palmer S, Klosky J, Deptula D, Thomas J, Cohen R (2005). A new measure of children's optimism and pessimism: the youth life orientation test. J Child Psychol Psychiatry.

[31] Bamford C, Lagattuta KH (2012). Looking on the bright side: children's knowledge about the benefits of positive versus negative thinking. Child Dev.

[32] Crothers L, Levinson E (2004). Assessment of Bullying: A Review of Methods and Instruments. Journal of Counseling & Development.

[33] Perren S, Alsaker FD (2006). Social behavior and peer relationships of victims, bully-victims, and bullies in kindergarten. J Child Psychol Psychiatry.

[34] Verlinden M, Veenstra R, Ringoot AP, Jansen PW, Raat H, Hofman A, Jaddoe VWV, Verhulst FC, Tiemeier H (2014). Detecting bullying in early elementary school with a computerized peer-nomination instrument. Psychol Assess.

[35] Mayeux L, Underwood MK, Risser SD (2007). Perspectives on the Ethics of Sociometric Research with Children: How Children, Peers, and Teachers Help to Inform the Debate. Merrill-Palmer Quarterly.

[36] Achenbach T (1991). Manual for the Teachers Report Form and 1991 Profile.

[37] de Winter AF, Oldehinkel AJ, Veenstra R, Brunnekreef JA, Verhulst FC, Ormel J (2005). Evaluation of non-response bias in mental health determinants and outcomes in a large sample of pre-adolescents. Eur J Epidemiol.

[38] Boivin M, Brendgen M, Dionne G, Dubois L, Pérusse D, Robaey P, Tremblay RE, Vitaro F (2013). The Quebec Newborn Twin Study into adolescence: 15 years later. Twin Res Hum Genet.

[39] Humphreys KL, Lee SS, Tottenham N (2013). Not all risk taking behavior is bad: Associative sensitivity predicts learning during risk taking among high sensation seekers. Pers Individ Dif.

[40] TRAILS Next.

