# Supplementary material for: Reward Responsiveness, Optimism, and Social and Mental Functioning in Children Aged 6-7: Protocol of a Cross-Sectional Pilot Study
Source: JMIR Res Protoc. 2020 Sep 24;9(9):e18902. doi: 10.2196/18902 (PMC7545321; doi:10.2196/18902)
Supplement: Multimedia Appendix 1 [file resprot_v9i9e18902_app1.pdf]

Reward Responsiveness, Optimism, and Social and Mental Functioning in Children Aged 6-7: Protocol of a Cross-Sectional Pilot Study

Charlotte Vrijen*, Mégane Alice Ackermans¶, Anna Bosma¶ & Tina Kretschmer

* Corresponding author: c.vrijen@rug.nl

¶ Both co-authors contributed equally to this paper

Multimedia Appendix 1. All child questionnaire items used in the present study, including the Dutch translations and original formulations.

Table S1

Child Questionnaires and Translations

No.	Original item	Dutch adapted item	Translation adapted item	Response options
**The pleasure scale** (based on [1])				
1	Someone that you like very much calls you and asks you to come and play with him/her.	Iemand die je heel aardig vindt vraagt of je mee gaat spelen.	Someone that you like very much asks you to play with him/her.	(How do you feel when this happens to you?) very happy – happy – does not really matter
2	a. You sit eating your favorite meal, which someone has cooked for you. b. You come home from school and smell your favorite cookies baking in the oven.	Je komt thuis en je hoort dat jullie je lievelingseten gaan eten.	You come home and hear you are going to eat your favorite meal.	
3	Your teacher makes you “King/Queen for a day” because you are such a great student.	Je juf/meester zegt aan het eind van de dag dat je het heel goed gedaan hebt vandaag.	At the end of the day, your teacher says that you did very well today.	
4	Someone buys you the new toy you’ve been asking for since last year.	Je krijgt op je verjaardag het speelgoed dat je al heel lang wilt hebben.	For your birthday you get the gift you’ve wanted for a long time.	
5	While playing your favorite video game, you beat the high score listed on the game board.	Je speelt je lievelingscomputerspel en haalt de hoogste score.	You are playing your favorite computer game and get the highest score.	
6	You are at a party with all your friends, good music and lots of good food.	Je gaat naar het verjaardagsfeestje van een goed vriendje en al je vriendjes en vriendinnetjes zijn er.	You go to a good friend’s party and all your friends are there.	
7	Your mother tells you how neatly you keep your room.	Je papa of mama zegt dat je heel goed zelf je speelgoed hebt opgeruimd.	Your parents tell you that you cleaned up your toys very well by yourself.	
8	Your friend tells you that “you are the best friend he/she ever had.”	Je vriendje/vriendinnetje zegt tegen je dat je zijn/haar allerbeste vriend(in) bent.	Your friend tells you that you are his/her best friend.	

**Optimism/pessimism** (based on [2,3])				
1	When I am not sure what will happen next, I usually think it will be good.	Als ik niet weet wat we gaan doen in de klas, denk ik dat het iets leuks is.	When I am not sure what will happen next at school, I usually think it will be good.	(How often do you think this?)
2	When things are good, I think something will go wrong.	Als ik leuk aan het spelen ben, dan denk ik vaak dat er iets fout gaat.	When I am having fun playing, I think something will go wrong.	never – sometimes – often – always
3	I usually think I will have a good day	Als ik ´s ochtends wakker wordt dan denk ik meestal dat het een leuke dag wordt.	When I wake up, I usually think today will be a good day.	
4	Overall, I think more good things will happen to me than bad things.	Er gebeuren vaker leuke dingen bij mij dan vervelende dingen.	Overall, more good things happen to me than bad things.	
5	Each day I think bad things will happen.	Ik denk vaak dat er morgen iets vervelends gaat gebeuren.	I often think bad things will happen tomorrow.	
6	When things are bad, I think they will get better.	Als iets niet goed gaat denk ik dat het straks beter gaat.	When things go wrong, I think they will get better.	
**Social Experiences**				
1		Met welke kinderen uit je klas vind je het leuk om te spelen?	Which classmates do you like to play with?	choose from all classmates
2		Met welke kinderen uit je klas speel je liever niet?	Which classmates do you not like to play with?	
3		Zijn er kinderen in de klas die andere kinderen uitschelden of slaan? Wie zijn dat?	Are there classmates who call other children names or hit other children? Who are they?	
4		Zijn er kinderen in de klas die worden uitgescholden of geslagen? Wie zijn dat?	Are there classmates who are called names or hit by other children? Who are they?	

Note. The following items were part of a pilot with five parents and their children, but were excluded from the pleasure scale afterwards because we wanted to reduce the number of items: ‘You win the first prize in a contest at school’ (reason: overlap with current item 5) and ‘Your parents tuck you in and give you a kiss good night’ (reason: we wanted to exclude one more item and thought that the other items better represented the different domains). The following items were excluded from the optimism/pessimism questionnaire after the pilot: ‘Usually I don’t think good things will happen to me’ (reason: four out of five parents reported that the item was too difficult for their child) and ‘Each day I look forward to having a lot of fun’ (reason: overlap with current item 3).

References

1 Kazdin AE. Evaluation of the Pleasure Scale in the Assessment of Anhedonia in Children. *Journal of the American Academy of Child & Adolescent Psychiatry* 1989;**28**:364–72. doi:10.1097/00004583-198905000-00010

2 Bamford C, Lagattuta KH. Looking on the Bright Side: Children's Knowledge About the Benefits of Positive Versus Negative Thinking. *Child Development* 2012;**83**:667–82. doi:10.1111/j.1467-8624.2011.01706.x
3 Ey S, Hadley W, Allen DN, *et al.* A new measure of children's optimism and pessimism: the youth life orientation test. *Journal of Child Psychology and Psychiatry* 2005;**46**:548–58. doi:10.1111/j.1469-7610.2004.00372.x
