# Supplementary material for: Reward Responsiveness, Optimism, and Social and Mental Functioning in Children Aged 6-7: Protocol of a Cross-Sectional Pilot Study
Source: JMIR Res Protoc. 2020 Sep 24;9(9):e18902. doi: 10.2196/18902 (PMC7545321; doi:10.2196/18902)
Supplement: Multimedia Appendix 2 [file resprot_v9i9e18902_app2.pdf]

Reward Responsiveness, Optimism, and Social and Mental Functioning in Children Aged 6-7: Protocol of a Cross-Sectional Pilot Study

Charlotte Vrijen*, Mégane Alice Ackermans¶, Anna Bosma¶ & Tina Kretschmer

* Corresponding author: c.vrijen@rug.nl

¶ Both co-authors contributed equally to this paper

Multimedia Appendix 2. All teacher questionnaire items used in the present study, including the Dutch translations and original formulations.

Table S2

Teacher Questionnaires and Translations

No.	Original Dutch item	Translation item	Response options
**Mental functioning of the child** [1]			
1	**Teruggetrokken gedrag.** Is liever alleen dan met anderen, is teruggetrokken, is te weinig in contact met anderen, is gesloten, weigert te praten, is te weinig actief of toont gebrek aan energie, kijkt met lege of wezenloze blik, zit te mokken.	**Withdrawal.** Prefers to be alone than with others, is withdrawn, has too little contact with others, is closed, refuses to speak, is not active or lacks energy, has a blank stare, sulks.	does not apply – rarely applies – somewhat applies – does apply – definitely applies
2	**Angstig/depressief.** Voelt zich eenzaam, huilt veel, vindt dat hij/zij perfect moet zijn, is bang om fouten te maken, voelt zich onbemind, voelt zich waardeloos of minderwaardig, is zenuwachtig of gespannen, is te bang, heeft veel last van schuldgevoel, schaamt zich gauw, is achterdochtig, kan niet tegen kritiek, is ongelukkig, verdrietig of gedeprimeerd, maakt zich zorgen.	**Anxious/depressed.** Feels lonely, cries a lot, believes he/she should be perfect, is afraid of making mistakes, feels unloved, feels unworthy, is nervous or tense, is too afraid, often feels guilty, is easily embarrassed, is suspicious, cannot handle criticism, is unhappy, sad or depressed, is worried.	
3	**Sociale problemen.** Gedraagt zich te jong, is te afhankelijk, kan niet opschieten met andere leerlingen, heeft het gevoel dat anderen het op hem/haar gemunt hebben, wordt veel geplaagd, andere leerlingen mogen hem/haar niet, gaat liever om met jongere kinderen.	**Social problems.** Acts too young, is too dependent, does not get along with classmates, feels targeted by others, is often teased, is not liked by classmates, prefers to interact with younger children.	

4	**Denkproblemen.** Kan bepaalde gedachten niet uit zijn/haar hoofd zetten, is bang voor bepaalde dieren, situaties of plaatsen, herhaalt alsmaar bepaalde handelingen, hoort geluiden of stemmen die er niet zijn, ziet dingen die er niet zijn, doet vreemd, heeft vreemde gedachten, verwondt zichzelf opzettelijk.	**Cognitive problems.** Cannot get certain thoughts out of his/her head, is afraid of certain animals, situations or places, constantly repeats certain actions, hears sounds or voices that are not there, sees things that are not there, behaves strangely, has strange thoughts, purposely harms him/herself.	
5	**Aandachtsproblemen.** Maakt niet af waar hij/zij mee begint, kan zich niet concentreren of lang de aandacht bij iets houden, is in de war, zit te dagdromen, heeft moeilijkheden met leren, is onhandig of slecht gecoördineerd, is onoplettend, makkelijk afgeleid, presteert beneden eigen niveau, voert opgedragen taken niet uit.	**Attentional problems.** Does not finish what he/she started, cannot concentrate or maintain concentration for long, is confused, daydreams, has difficulties with schoolwork, is clumsy or has poor coordination, is inattentive, is easily distracted, performs below his/her abilities, does not complete assigned tasks.	
6	**Activiteit/impulsiviteit.** Bromt of maakt andere vreemde geluiden tijdens de les, kan niet stilzitten, is onrustig of over-actief, zit te wiebelen of te friemelen, vindt het moeilijk om aanwijzingen op te volgen, is impulsief, handelt zonder nadenken, werkt slordig.	**Activity/impulsivity.** Hums or makes other strange noises during class, cannot sit still, is agitated or hyperactive, wiggles or fidgets, has difficulties following instructions, is impulsive, acts without thinking, works carelessly.	
7	**Agressief gedrag.** Spreekt tegen of maakt ruzie, is uitdagend, is brutaal, schept op, is wreed, is pesterig, eist veel aandacht op, vernielt spullen van zichzelf of anderen, is ongehoorzaam, stoort andere leerlingen, veroorzaakt veel onrust in de klas, is jaloers, vecht veel, valt anderen lichamelijk aan, schreeuwt of gilt veel, is explosief of onvoorspelbaar, is snel gefrustreerd, is koppig of prikkelbaar, verandert snel van stemming, plaagt veel, heeft driftbuien of is snel driftig, bedreigt andere mensen.	**Aggressive behavior.** Contradicts others or argues, provokes others, is rude, brags, is cruel, bullies others, demands a lot of attention, destroys objects, is disobedient, bothers other students, causes trouble in class, is jealous, fights a lot, physically attacks others, screams or yells a lot, is explosive or unpredictable, is easily frustrated, is stubborn or irritable, has mood swings, teases a lot, throws tantrums or has a short temper, threatens others.	
8	**Delinquent gedrag.** Lijkt zich niet schuldig te voelen als hij/zij zich misdragen heeft, gaat om met jongens/meisjes die in moeilijkheden raken, liegt of bedriegt, is liever samen met oudere jongens of meisjes, steelt, vloekt of gebruikt schuttingtaal.	**Delinquent behavior.** Does not appear to feel guilty when he/she misbehaves, interacts with boys/girls that get into trouble, lies or deceives, prefers to interact with older boys or girls, swears or uses foul language.	
**Social functioning of the child** [2]			
1	Hoe vaak heeft dit kind het afgelopen half jaar andere kinderen uitgelachen of belachelijk gemaakt?	In the past six months, how often has this child made fun of other children?	never – sometimes – often
2	Hoe vaak heeft dit kind het afgelopen half jaar andere kinderen geslagen of geduwd?	In the past six months, how often has this child hit or pushed other children?	
3	Hoe vaak heeft dit kind het afgelopen half jaar andere kinderen uitgescholden?	In the past six months, how often has this child called other children names?	

4	Hoe vaak heeft dit kind het afgelopen half jaar andere kinderen buitengesloten?	In the past six months, how often has this child excluded other children?	
5	Hoe vaak is dit kind het afgelopen half jaar door andere kinderen uitgelachen of belachelijk gemaakt?	In the past six months, how often was this child made fun of by other children?	
6	Hoe vaak is dit kind het afgelopen half jaar door andere kinderen geslagen of geduwd?	In the past six months, how often was this child hit or pushed by other children?	
7	Hoe vaak is dit kind het afgelopen half jaar door andere kinderen uitgescholden?	In the past six months, how often was this child called names by other children?	
8	Hoe vaak is dit kind het afgelopen half jaar door andere kinderen buitengesloten?	In the past six months, how often was this child excluded by other children?	
9	Geef voor uw leerling aan in hoeverre de volgende uitspraak van toepassing is: Andere kinderen vinden het leuk om met dit kind te spelen.	Please indicate to what extent the following statement applies to the child: Other children like to play with the child.	does not apply – rarely applies – somewhat applies – does apply – definitely applies

References

1 De Winter AF, Oldehinkel AJ, Veenstra R, *et al.* Evaluation of non-response bias in mental health determinants and outcomes in a large sample of pre-adolescents. *Eur J Epidemiol* 2005;**20**:173–81. doi:10.1007/s10654-004-4948-6

2 Boivin M, Brendgen M, Dionne G, *et al.* The Quebec Newborn Twin Study Into Adolescence: 15 Years Later. *Twin Research and Human Genetics* 2013;**16**:64–9. doi:10.1017/thg.2012.129
